# Supplementary material for: A comprehensive characterization of PncA polymorphisms that confer resistance to pyrazinamide
Source: Nat Commun. 2017 Sep 19;8:588. doi: 10.1038/s41467-017-00721-2 (PMC5605632; doi:10.1038/s41467-017-00721-2)
Supplement: Supplementary file 2 — Supplementary Information [file 41467_2017_721_MOESM2_ESM.pdf]

Description of Supplementary Files

File Name: Supplementary Information
Description: Supplementary Figures, Supplementary Tables and Supplementary References

File Name: Supplementary Data 1
Description: pncA single-nucleotide polymorphisms statistically enriched or depleted after pyrazinamide selection. Under-represented and discrepant (enriched and depleted after pyrazinamide selection) singlenucleotide polymorphisms were removed from the analysis.

File Name: Supplementary Data 2
Description: PncA amino acid substitutions catalogued resistant or susceptible after pyrazinamide selection in vitro or after infection in mice. Under-represented and discrepant (enriched and depleted after pyrazinamide selection) amino acid substitutions were removed from the analysis. Catalogue is the amino acid substitutions collated from the pyrazinamide susceptibilities in vitro and after infection in mice. Mutations statistically enriched (E) after pyrazinamide selection in vitro and/or in mice. Mutations statistically depleted (D) after pyrazinamide selection in vitro and/or in mice. Mutations unselected (U) after pyrazinamide treatment either in vitro or infection in mice. Resistant mutations as reported in Miotto et. al32 and Walker et. al47 are annotated.

File Name: Peer Review File

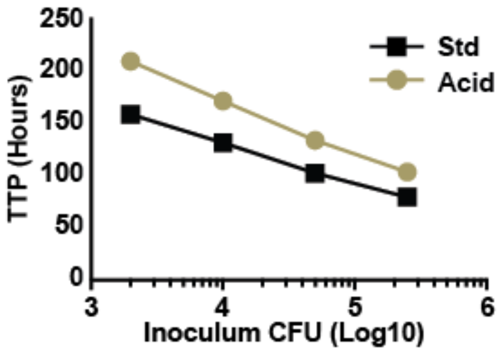

Supplementary Figure 1 Relationship of inoculum (colony forming units, CFU) to time-to-positivity (TTP, hours) in the BD BACTEC MGIT system using pH 5.9 (Acid) and pH 6.8 (Std) media. Mean and standard-deviations of three biological replicates are shown.

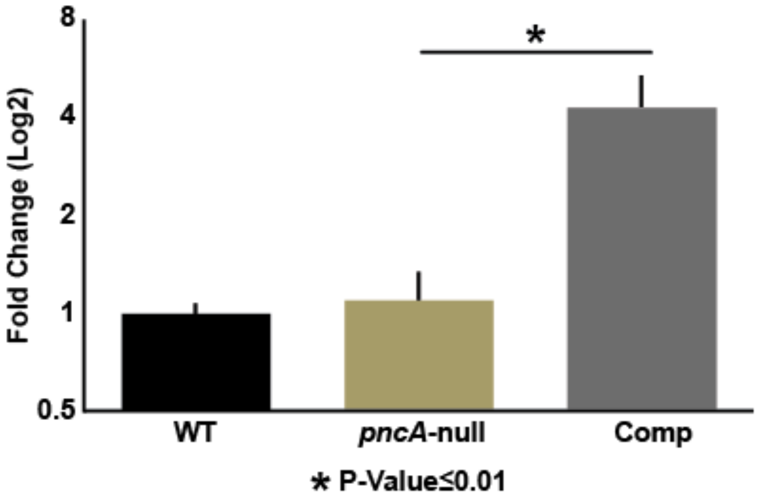

Supplementary Figure 2 Complementation of the *pncA*-null strain results in over-expression of *pncA* relative to the isogenic wild type (WT) strain as measured by RT-qPCR.

The *pncA*-null strain was obtained from Boshoff et al.[38] and contains an insertionally inactivated *pncA*::*hyg* allele. Errors bars represent the standard deviations derived from the propagation of error using the quotient of the coefficient of variation. * corresponds to P-value ≤ 0.01 (*t*-test, two-sided).

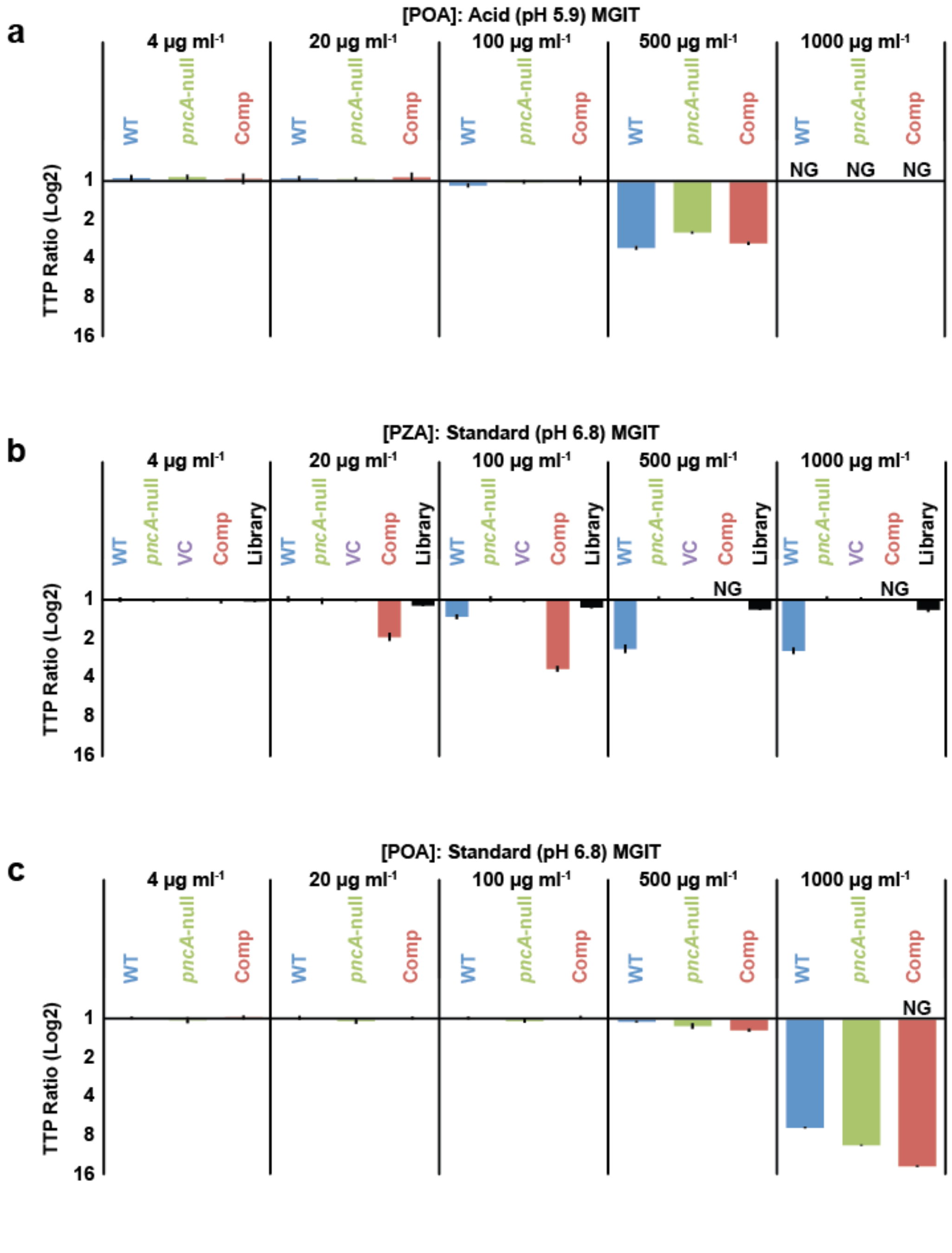

Supplementary Figure 3 Pyrazinamide (PZA) and pyrazinoic acid (POA) growth inhibition in four isogenic control strains, WT, *pncA*-null, Comp, and vector control (VC), and the *pncA* mutant library.

(**a**) Growth inhibition with pyrazinoic acid (POA) in pH 5.9 (Acid) media. (**b**) Growth inhibition with pyrazinamide (PZA) in pH 6.8 (Standard) media. (**c**) Growth inhibition with pyrazinoic acid (POA) in pH 6.8 (Standard) media. Time-to-positivity (TTP) ratio is the average ratio of the time-to-positivity of the test condition to the no drug control. A minimum of three biological replicates per strain per test condition was performed. Errors bars represent the standard deviations derived from the propagation of error using the quotient of the coefficient of variation.

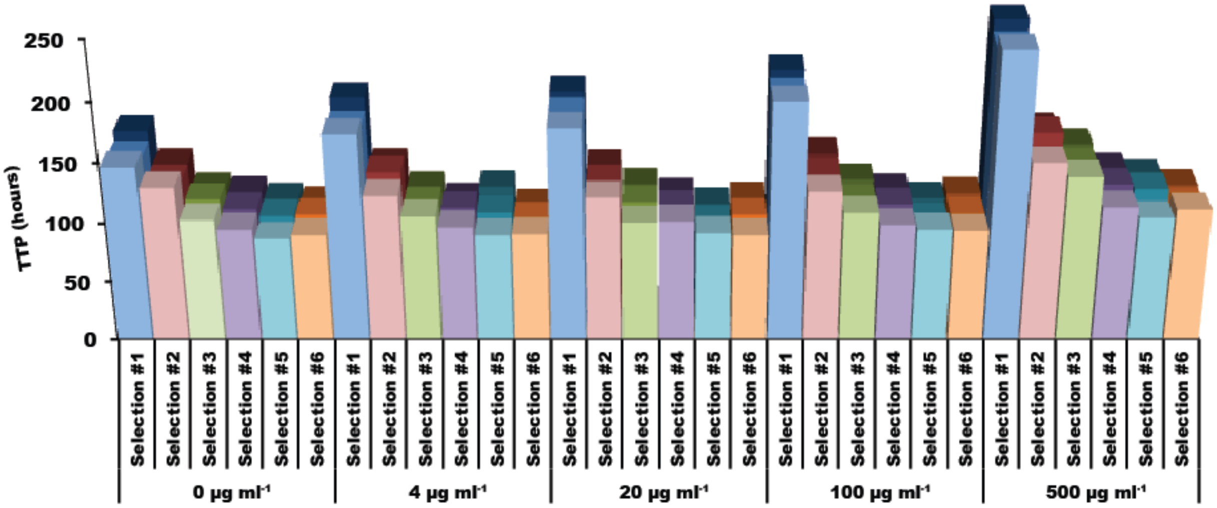

Supplementary Figure 4 Time-to-positivity (TTP; hours) of each of three biological replicates for each round of *in vitro* selection.

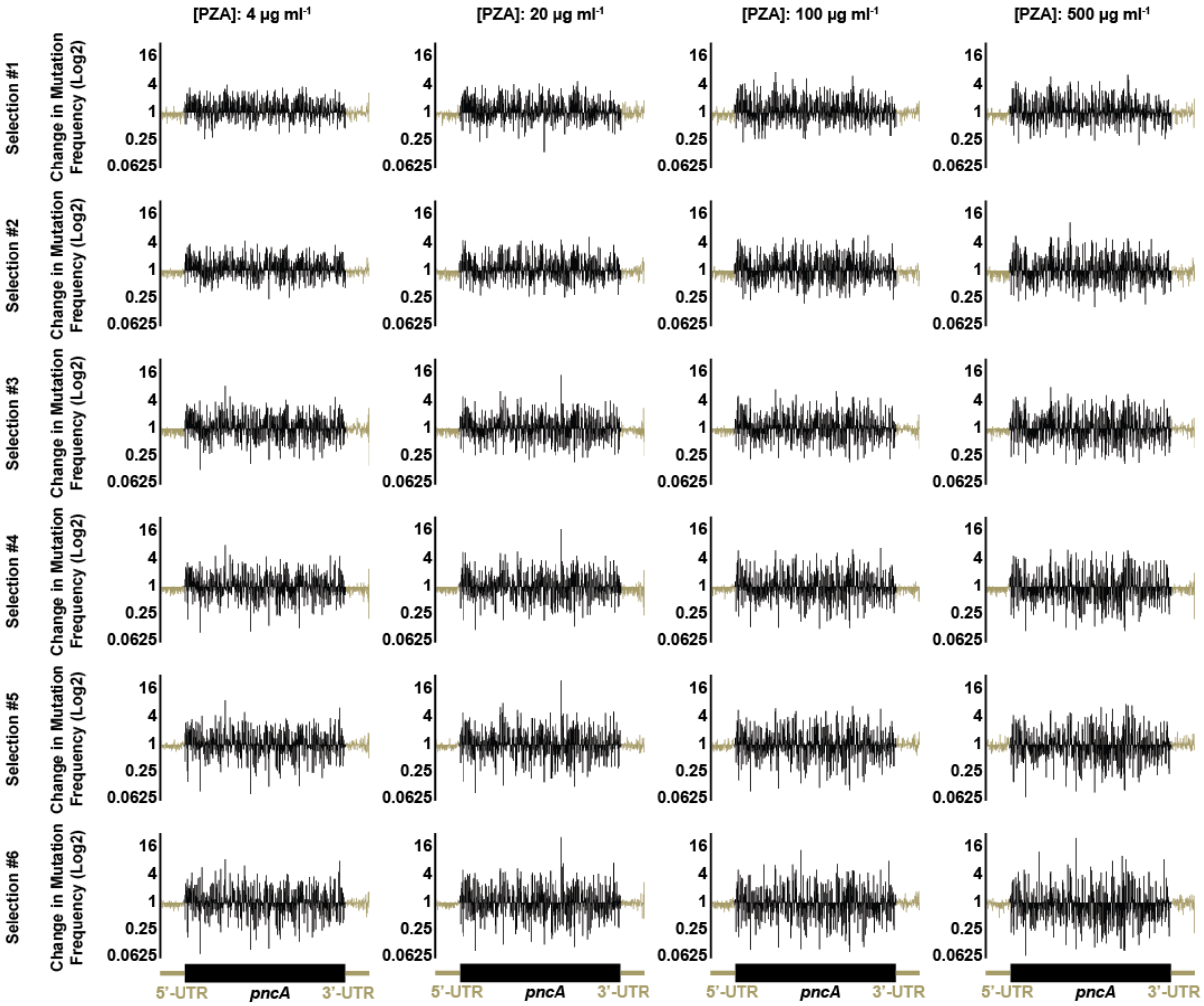

Supplementary Figure 5 Positive and negative selection occurs at individual nucleotides as a result of *in vitro* pyrazinamide selection.

The mean *pncA* single-nucleotide polymorphism frequency (%) from three biological replicates at each nucleotide (relative to the no-drug control of the same selection round) across all 24 pyrazinamide selections.

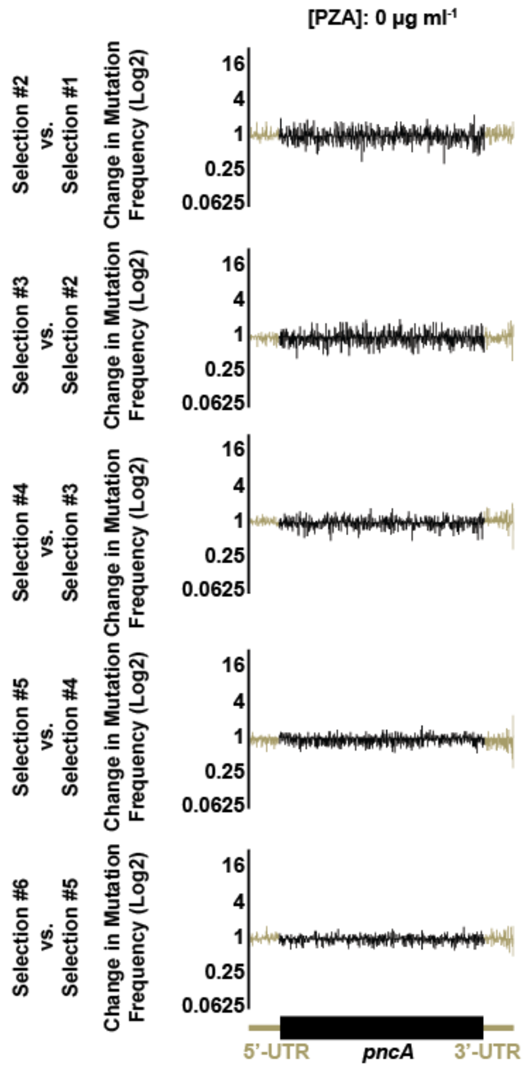

Supplementary Figure 6 No evidence of selection is observed during six rounds of *in vitro* selection without pyrazinamide.

The mean *pncA* single-nucleotide polymorphism frequency (%) from three biological replicates at each nucleotide relative to the no-drug control of the preceding selection round.

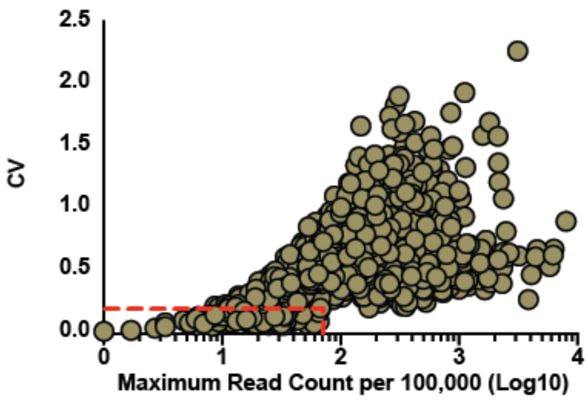

Supplementary Figure 7 Complexity of the *pncA* library determined by Illumina amplicon sequencing.

Low abundance single-nucleotide polymorphisms (SNPs), defined as a maximum normalized read count of <70 per 100,000 reads, showing no evidence of selection, defined as a coefficient of variation (CV) of <0.2 between rounds of selection, were discarded as under-represented (dashed red line). Maximum read count is the highest read count frequency of a given mutation that occurred in any round of in vitro selection.

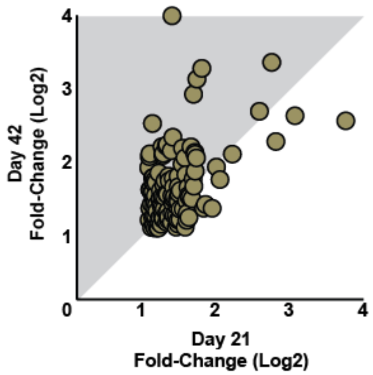

Supplementary Figure 8 Pyrazinamide selection after 42 days infection in mice is ongoing.

Correlation between the fold-change (log2) in single-nucleotide polymorphisms enriched after both 21 and 42 days of pyrazinamide selection in mice.

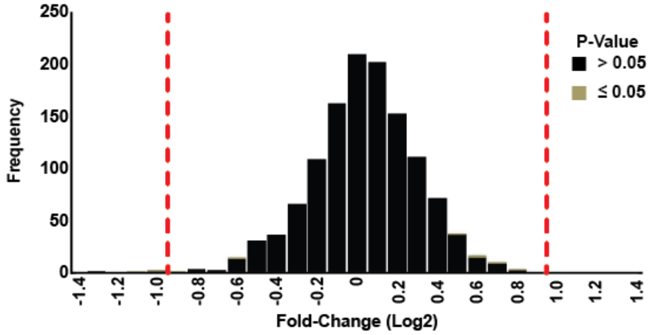

Supplementary Figure 9 PncA substitutions have minimal fitness cost in mock treated mice.

The frequency of amino acid substitutions after 42 days of mock treatment in mice with a given fold-change relative to the implantation library. Black is the proportion of amino acid substitutions with a p-value > 0.05. Brown is the proportion of amino acid substitutions with a p-value ≤ 0.05. Dashed red lines demarcate ≥ 2-fold change. P-values were calculated by fitting a negative binomial distribution to each replicate of normalized read counts in each condition, using empirical Bayes estimation of variances, and comparing the differences in scaled means using a Wald test, as described in the methods. The resulting p-values were adjusted for multiple comparisons using the Benjamini-Hochberg procedure.

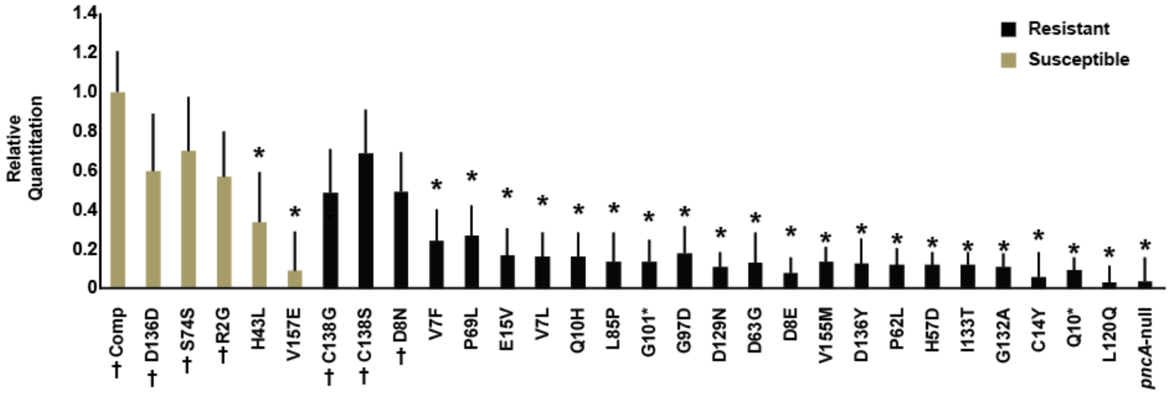

Supplementary Figure 10 PncA protein abundance.
Quantitation of PncA protein abundance relative to DnaK in catalogued pyrazinamide susceptible (brown) and resistant (black) isolates determined using mass spectrometry. Protein abundance in each mutant strain is relative to the Comp strain. * corresponds to a P-Value ≤ 0.01 (*t*-test, two-sided). † corresponds to isolates tested for enzymatic activity. The mean fold-change of three biological replicates for each mutant relative to the mean of 10 biological replicates for Comp is shown. Errors bars represent the standard deviations derived from the propagation of error using the quotient of the coefficient of variation.

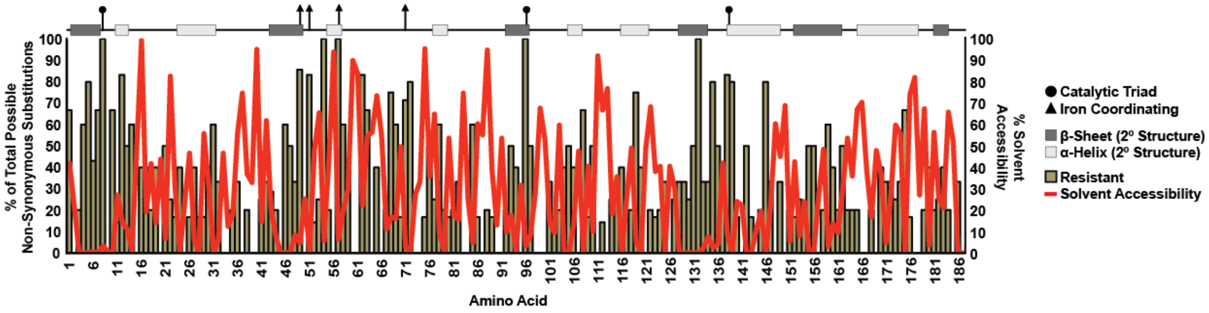

Supplementary Figure 11 Pyrazinamide resistant amino acid substitutions have low solvent accessibility.

The proportion (%) of non-synonymous amino acid substitutions represented in the *pncA* library that were pyrazinamide resistant at each PncA amino acid (brown bars) and the solvent accessibility (%) of each wild type amino acid (red line). Amino acids corresponding to the catalytic triad are marked with a circle. Amino acids responsible for iron coordination are marked with a triangle.

	Amino Acid Substitution	*in vitro* Selection	Mouse Selection	MIC ($\mu g\ ml^{-1}$)
	Comp (WT)	NA	NA	1
	ΔpncA	NA	NA	>500
Synonymous	S74S	U	U	1
	V109V	D	D	1
	D136D	D	D	1
	I6I	U	D	4
	D8D	U	D	4
	S18S	D	D	4
	V93V	D	U	4
	A102A	U	U	4
	G108G	D	D	4
	V131V	U	D	4
	A134A	D	D	4
Non-Synonymous	H43L	U	D	1
	G55R	U	U	1
	S104N	U	U	1
	R2G	U	U	4
	V7I	D	U	4
	V9M	U	D	4
	D40E	D	D	4
	S67W	D	U	4
	C72S	D	D	4
	F81I	U	U	4
	F106L	D	U	4
	Q122R	U	U	4
	E144K	D	D	4
	V147L	U	D	4
	V157E	U	U	4
	R2W	U	U	20
	E15V	U	U	20
	P62L	E	E	20
	D63G	E	E	20
	D129N	U	E	20
	I133T	U	U	20
	V155M	U	E	20
	V7L	E	E	100
	G97D	E	E	100
	D8E	E	E	500
	C14Y	E	E	500
	L85P	E	E	500
	V7F	E	E	>500
	D8N	E	E	>500
	Q10H	E	E	>500
	Q10*	E	E	>500
	H57D	E	E	>500
	P69L	U	E	>500
	G101*	E	E	>500
	L120Q	U	E	>500
	G132A	E	E	>500
	D136Y	E	E	>500
	C138G	E	E	>500
	C138S	E	E	>500

Supplementary Table 1 Minimum inhibitory concentration (MIC) validates pyrazinamide susceptibilities determined after selection *in vitro* and infection in mice. Amino acid substitutions enriched (E), depleted (D) or showing no selection (U) after pyrazinamide treatment *in vitro* or in mice. MIC corresponds to the minimum inhibitory concentration determined using the BD BACTEC MGIT system. NA corresponds to non-applicable.

Name	Number	Description	Source
WT (H37Rv)	sAY101	ATCC 27294	This study
pncA-null	sAY251	H37Rv_pncA::HygR	Supplementary Ref. 1
Comp (WT)	sAY245	sAY251 containing pAY108	This study
Vector Control (VC)	sAY257	sAY251 containing pAY111	This study
pncA Mutant Library	sAY260	sAY251 containing pAY230	This study

Supplementary Table 2 List of strains used in this study.

Plasmid	Description	Source
pAY59	ZeoR, L5 integrating vector	This study
pAY107	ZeoR, L5 integrating vector containing WT pncA	This study
pAY108	ZeoR, L5 integrating vector with WT pncA constitutively expressed from the MOP promoter	This study
pAY111	ZeoR, L5 integrating vector with Emerald GFP	This study
pAY112	ZeoR, L5 integrating vector with Emerald GFP constitutively expressed by the MOP promoter	This study
pAY230	ZeoR, L5 integrating vector with MOP expressed pncA mutagenesis Ibrary	This study

Supplementary Table 3 List of plasmids used in this study.

Primers	5' --> 3'
ANY_P55	AAATTTACATGTTCTAGAGCTAGGAGCTGCAAACCAACTCGACGCT
ANY_P57	AAATTTATCGATGCGGCCGCATGCGGGCGTTGATCATCGTCGA
ANY_P58	TGGCAGTCGATCGTACGCTAGTT
ANY_P59	GAGCCTATGGAAAAACGCCAGCA
ANY_P147	AAATTTGCGGCCGCATGAGCAAGGGCGAGGAGCT
ANY_P148	GGTGGTACATGTTCTAGACTTGTAGAGCTCGTCCATGCC
ANY_P149	AAATTTATCGATCCCCAGGCTTGACACTTTATG
ANY_P150	GGTGGTGCGGCCGCTCTCCTGCTGGATCCGAATTGTGAGC
ANY_P153	GTGGACGTGCGGCCGCATG
ANY_P154	GGCCTTTTGCTCACATGTTCTAGAGCTA
ANY_P158	CGCCACCTCTGACTTGAGCG
ANY_P159	GGGAGTTGCAGCGGTCGG
KM_P35	GGCACACCGGACTATTCCTC
KM_P36	CCCTTGTAGAACACCGCCTC
sigA_F	CCTACGCTACGTGGTGGATT
sigA_R	TGGATTTCCAGCACCTTCTC

Supplementary Table 4 List of primers used in this study.

Name	Peptide
pncA-1	AISDYLAEAADYHHVVATK
pncA-2	GAYTGAYSGFEGVDENGTPLLNWLR
pncA-3	GVDEVDVVGIATDHCVR
rpoB	VNPFGFIETPYR
dnaK	LLGSFELTGIPPAPR

Supplementary Table 5 List of peptides used for proteomic analysis in this study.

SUPPLEMENTARY REFERENCES

1. Boshoff HI, Mizrahi V. Expression of Mycobacterium smegmatis pyrazinamidase in Mycobacterium tuberculosis confers hypersensitivity to pyrazinamide and related amides. *Journal of bacteriology* **182**, 5479-5485 (2000).
